# Extended Cost-Effectiveness Analysis for Health Policy Assessment: A Tutorial

**DOI:** 10.1007/s40273-016-0414-z

**Published:** 2016-07-04

**Authors:** Stéphane Verguet, Jane J. Kim, Dean T. Jamison

**Affiliations:** 1 Department of Global Health and Population, Harvard T.H. Chan School of Public Health, 665 Huntington Avenue, Boston, MA 02115 USA; 2 Department of Health Policy and Management, Harvard T.H. Chan School of Public Health, Boston, MA USA; 3 Global Health Sciences, University of California, San Francisco, CA USA; 4 Department of Global Health, University of Washington, Seattle, WA USA

## Abstract

Health policy instruments such as the public financing of health technologies (e.g., new drugs, vaccines) entail consequences in multiple domains. Fundamentally, public health policies aim at increasing the uptake of effective and efficient interventions and at subsequently leading to better health benefits (e.g., premature mortality and morbidity averted). In addition, public health policies can provide non-health benefits in addition to the sole well-being of populations and beyond the health sector. For instance, public policies such as social and health insurance programs can prevent illness-related impoverishment and procure financial risk protection. Furthermore, public policies can improve the distribution of health in the population and promote the equalization of health among individuals. Extended cost-effectiveness analysis was developed to address health policy assessment, specifically to evaluate the health and financial consequences of public policies in four domains: (1) the health gains; (2) the financial risk protection benefits; (3) the total costs to the policy makers; and (4) the distributional benefits. Here, we present a tutorial that describes both the intent of extended cost-effectiveness analysis and its keys to allow easy implementation for health policy assessment.

## Key Points for Decision Markers

**Table:** 

Extended cost-effectiveness analysis (ECEA) fundamentally builds on cost-effectiveness analysis and provides quantitative methods for health policy assessment. It examines public policies, whether health or inter-sectoral policies, and policy instruments that have an impact on the health of populations.
ECEA further assesses the health and financial consequences of policies, including financial risk protection and disaggregated outcomes per population stratum of interest.
The ECEA approach permits the inclusion of non-health benefits (financial risk protection) and distributional consequences or equity in the economic evaluation of health policies. It enables the consideration of key criteria into the resource allocation problem and into the design of the health benefits package.

## Background

Economic evaluations for health, cost-effectiveness analyses, or CEAs, have essentially focused on quantifying the health gains per given expenditure on a health intervention [1–3]. In this accounting exercise, the health benefits can include directly measurable outcomes such as deaths averted or disease cases averted, or can rely on constructed metrics such as quality-adjusted life-years (QALYs) gained or disability-adjusted life-years (DALYs) averted. The research analyst often expresses incremental cost-effectiveness ratios as dollars per death averted, dollars per DALY averted, or dollars per QALY gained. As a result, CEA has been largely dedicated to the economic evaluation of health interventions and in particular of new technologies and drugs (e.g., vaccines, cancer drugs), often to identify the ‘best buys’ and ‘magic bullets’, which ultimately policy makers and governmental bodies may promote in a publicly financed benefits package.

Yet, many have argued that CEA in health should move towards explicit consideration of the multiple dimensions and outcomes that ensue from health policies. For instance, financial risk protection (FRP, the attenuation or prevention of illness-related impoverishment) on the outcome side and the use of scarce health system capacity on the financial side should be included [3]. As an illustration, Kim and colleagues have analyzed the effect of health system constraints on optimal resource allocation in the context of cervical cancer screening [4], and Rheingans and others have examined the distributional impact of rotavirus immunization [5], in low- and middle-income countries.

Policy makers in ministries of health and ministries of finance rarely make their financial allocations solely based on CEA findings maximizing health gains per dollar spent, but rather examine a range of criteria before assigning resources within and outside the health sector. In many countries, equity and fairness dimensions are integral to the rationing process, and numerous tradeoffs stand out that can directly conflict with the sole efficiency figure of merit of cost per QALY gained as provided by a CEA [6]. Therefore, analytical frameworks attempting to capture the multiple criteria involved in the decision-making process have been developed [7]. A number of mathematical models have either focused on the explicit incorporation of some form of equity or population distributions into the resource allocation and decision-making problems [8–15], or have proposed to display analysis findings and outcomes in a disaggregated manner in the form of a dashboard [7, 16].

Within their primary mandate of improving or maintaining health, the World Health Organization characterized health systems as having three fundamental objectives: (1) to improve health and the distribution of health in the population; (2) to enhance responsiveness to the expectations of the population; and (3) to promote fairness in the financial contribution towards health [17]. After World War II in Western Europe, national health systems were designed with one of the fundamental intents being to prevent illness-related impoverishment and to provide FRP to the populations they serve. For example, the opening page of the United Kingdom’s National Health Service document of July 5, 1948 reads “there are no charges, except for a few special items. There are no insurance qualifications. But it is not a ‘charity.’ You are all paying for (the National Health Service), mainly as taxpayers, and it will relieve your money worries in times of illness” [18].

FRP objectives are critical in low- and middle-income countries where social insurance programs such as sick leave and unemployment coverage fail to cover large parts of the population. Protection from financial risks associated with healthcare expenses has emerged as a critical component of national health strategies in many countries. Indeed, out-of-pocket medical payments can lead to impoverishment with households choosing from among many coping strategies (e.g., borrowing from relatives, asset selling) to manage health-related expenses. Despite other financing mechanisms, household medical expenditures can often be ‘catastrophic’, defined as exceeding a certain fraction of total household expenditures [19]. Attention to illness-related impoverishment has been heightened with the recent institution of the Sustainable Development Goals (SDGs) by the United Nations in September 2015. SDG1 calls for “ending poverty in all its forms by 2030”; and SDG3, the health-related SDG, presents a sub-target on achieving “universal health coverage, including FRP and access to quality essential health services” [20].

Health inequalities are very substantial both across and within countries. Large variations in health outcomes across socioeconomic groups and the social determinants of health have long been demonstrated [21]. In the USA, for example, recent investigations have pointed to the wide differences in mortality outcomes and life expectancy at birth between states and racial groups [22, 23]. Inequalities in healthcare use also exist where often access to health services can be concentrated among the richer socioeconomic groups or well-off regions. For instance, in many low- and middle-income countries (e.g., Ethiopia), wealthy individuals can use essential health services two to three times more readily than poorer individuals [24].

One major objective of public policy making is to remove societies’ inequalities. In particular, health policy instruments fundamentally aim at increasing the uptake of effective and efficient interventions and subsequently lead to greater health benefits (e.g., premature mortality and morbidity averted). In addition, they can generate non-health benefits beyond the mere well-being of citizens and outside the health sector. For instance, public policies such as health insurance programs can prevent illness-related impoverishment and improve the distribution of health in populations towards the equalization of health among individuals. Specifically, extended CEA (ECEA) [25] was conceived for health policy assessment (HPA), i.e., to evaluate the health and financial consequences of health policies in four domains: (1) the health gains; (2) the FRP (prevention of illness-related impoverishment) benefits; (3) the total costs of the policy to the decision makers; and (4) the distributional (e.g., across socioeconomic groups) consequences. In this respect, ECEA highlights the returns on investment in the dimensions of equity and FRP, in addition to health benefits, per given budget expenditure on policy. This article proposes a practical tutorial to conduct ECEA for HPA.

## Extended Cost-Effectiveness Analysis: Praxis

### Health Policy Assessment

ECEA examines public policies, whether health or inter-sectoral policies, which have an impact on the health of populations, and is fundamentally concerned with policy instruments. Jamison [3] divides policy instruments into the following categories: mass education campaigns, legal and regulatory policies, financial policies (e.g., taxation, subsidies, user fees, and conditional cash transfers), engineering policies, and direct government provision of services or training. Examples can include: universal public finance (government financing of an intervention irrespective of whom is receiving it) or pro-poor public finance (government financing of an intervention targeting poorer segments of the population) for a package of immunizations; excise taxes on tobacco and alcohol products; a law enforcing a restriction on the salt content of breads; pedestrian pathways, speed bumps, and roundabouts.

The first step for the research analyst is thus to select a policy instrument of interest, denoted HP in what follows, for examination in a given population *P*. As often, the population *P* can be segmented and best interpreted through distinct population subgroups (denoted *P*_*k*_, with 1 ≤ *k* ≤ *n*). The indexation *k* may define a segmentation by socioeconomic status (e.g., per income quintile), by region or subnational geographical unit in a country (e.g., per province, state, county, district, municipality), by ethnicity or by sex, for example. Evidently, the definition and selection of the population subgroups *k* will depend on the specific questions, including equity and distributional issues, the analyst is posing. The second step is to specify an intervention provided by the policy instrument HP (e.g., vaccine for preventing rotavirus, treatment for stroke, prevention of road traffic injury), which will have a given coverage (i.e., Cov) and a given efficacy or effectiveness (i.e., Eff) towards prevention or treatment of the illness or condition. Enactment of the policy HP also entails a given net cost (i.e., *C*) to the implementer.

**Fig. 1:**
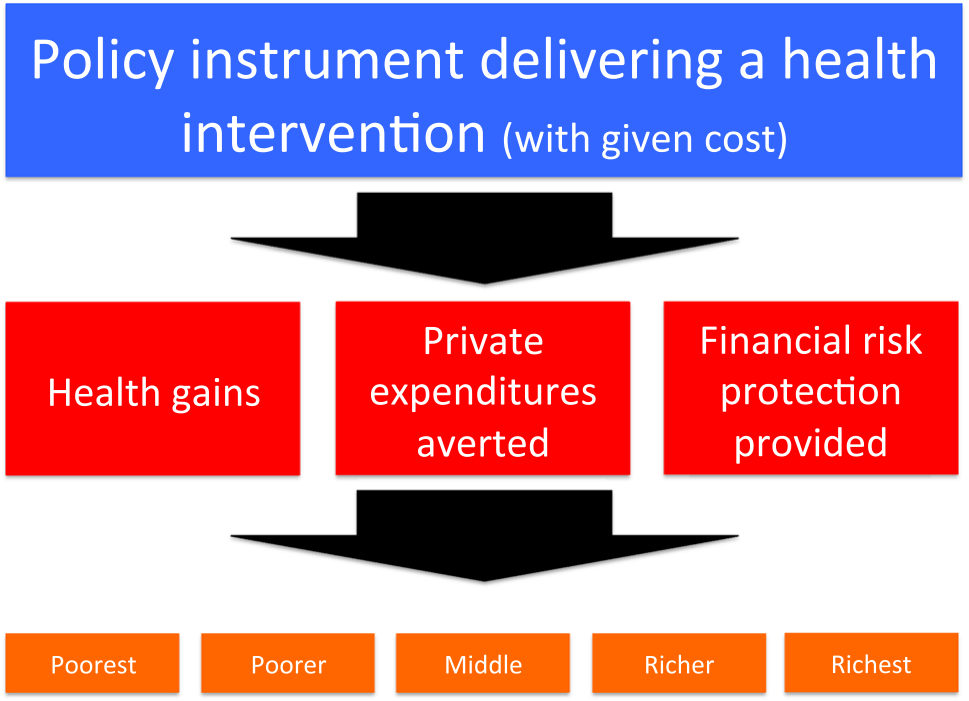
Conceptual structure of the extended cost-effectiveness analysis methodology where policy impact is estimated in four domains across distinct wealth strata of the country population: (1) health gains; (2) private expenditures averted; (3) prevention of illness-related impoverishment or financial risk protection provided; and (4) cost to the implementer

The purpose of the ECEA methodology is to quantitatively examine HPA. In pursuing HPA, ECEA explicitly quantifies the following four consequences per population subgroup $P_{k}$ for a given HP: (1) the health benefits procured by the policy; (2) the private expenditures and costs averted by the policy; (3) the FRP benefits provided by the policy; and (4) the total net costs of the policy (Fig. 1).

### Quantifying the Health Gains

**Fig. 2:**
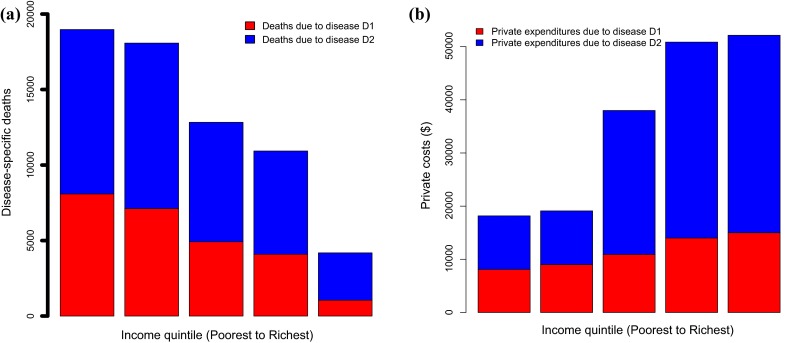
Estimated distribution across income quintiles in country ‘Land’ of: (**a**) deaths attributable to diseases D1 and D2 and (**b**) private expenditures (e.g. out-of-pocket direct medical costs) attributable to the treatment of diseases D1 and D2

The implementation of policy HP first leads to health gains distinctly accruing in each population subgroup *P*_*k*_. ECEA estimates the distributional health consequences (e.g., deaths averted, DALYs averted) per population stratum *P*_*k*_. To do so, we need to a priori know the distribution of health in the population. For instance, ECEA may require data inputs on the mortality attributable to a specific disease targeted by the policy HP per income quintile in the population (Fig. 2a). In other words, information on the relevant disease burden *D*_*k*_ per specific population stratum is necessary.

ECEA quantifies the health benefits procured by HP. These benefits depend notably on: the ex-ante disease burden (i.e., *D*_*k*_), the coverage of the intervention achieved by population subgroup (i.e., Cov_*k*_), and the effectiveness of the intervention, potentially per population subgroup (i.e., Eff_*k*_). Using a simple static disease model, the estimation of the health gains (i.e., *B*_*H*,*k*_) could be expressed in the following way:

1 $$ B_{H,k} = {\text{Eff}}_{k} *{\text{Cov}}_{k} * D_{k} . $$

Therefore, ECEA uses the exact same approach as a traditional CEA except that it examines the health gains procured across *k* population subgroups in lieu of 1 in the case of CEA. In other words, ECEA only studies one specific type of health equity impact (i.e., the distribution of health outcomes).

Consider for example the case of tuberculosis (TB) in country ‘Land’ with a population of 10 million (2 million per income quintile). In Land, TB incidence is 200 per 100,000 population per year on average, and is 400, 300, 200, 100, and 0 per 100,000 among the five country income quintiles, lowest to highest. Case fatality ratio from TB is 20 %, TB treatment is 80 % effective, and current treatment coverage is 50 % and uniform across each income quintile. Assume an increase in treatment coverage of 10 %, equally across the five income groups, through universal public finance (UPF). Using a simple static disease model, UPF for TB treatment in Land would lead to the following TB-related deaths averted per income quintile:

2 $$ B_{H} = 0.80*0.10*0.20*\left\{ {8000;6000;4000;2000;0} \right\} = \left\{ {128;96;64;32;0} \right\}. $$

UPF for TB treatment in Land would avert a total of 320 TB-related deaths, 40 % of which would occur among the poorest.

### Quantifying the Financial Consequences for Individuals

ECEA takes the perspective of the individuals affected by illness and examines the ensuing illness-related financial consequences they face. With the onset of illness, affected individuals’ financial burden can include: the direct payment of medical care out of pocket (denoted *c*_DM_), direct non-medical costs (most importantly transportation costs to seek care) out of pocket (denoted *c*_DNM_), and time and productivity losses, which can be translated into wages and income foregone (often named indirect or friction costs) (denoted *c*_I_). All such financial implications may vary by population subgroup (i.e., *c*_DM,*k*_, *c*_DNM,*k*_, *c*_I,*k*_). For example, some may visit private health facilities rather than public facilities, which may lead to a differential in out-of-pocket costs.

Similar to the quantification of health gains, the analyst must obtain prior information on the relevant financial burden to individuals tied to the specific illnesses and conditions addressed by the policy HP (Fig. 2b). In other words, we must obtain data inputs on the amount of private expenditures and costs, denoted *PE*_*k*_, incurred by individuals. Denote *i*_*k*_ and *u*_*k*_, the incidence of illness and the healthcare use for illness treatment (e.g., probability of seeking care conditional on having the disease) per population subgroup, respectively. The amount of private expenditures incurred by individuals in the population subgroup *P*_*k*_ could be expressed as:^1^

3 $$ {PE}_{k} = i_{k} *u_{k} *(c_{{{\text{DM}},k}} + c_{{{\text{DNM}},k}} ). $$

The implementation of policy HP may lead to the ‘crowding out’ of these individual private expenditures. That is to say HP can, partially or totally, remove *PE*_*k*_, leading to ‘private expenditures crowded out.’

ECEA further disaggregates what is named the ‘societal’ perspective in traditional economic evaluations [2], to precisely examine the perspective of individuals and estimate the amount of private expenditures (e.g., direct medical costs, direct non-medical costs, indirect costs) that could be averted by policy. Again, using a simple modeling approach, the private expenditures averted (i.e., *PE*_av,*k*_) could take the following mathematical expression:

4 $$ {PE}_{{{\text{av}},k}} = {\text{Cov}}_{k} *{PE}_{k} . $$

Consider the case of TB in Land, where 40 % of TB-infected individuals would purchase TB treatment entirely out of pocket at *c*_DM,*k*_ = $100. After UPF for TB treatment, they would no longer spend money out of pocket for TB treatment. The amount of private expenditures averted by UPF would then be:

5 $$ {PE}_{\text{av}} = 0.40*\left\{ {8000;6000;4000;2000;0} \right\}*100 = \$ \left\{ {320000;240000;160000;80000;0} \right\}. $$

In other words, UPF for TB treatment in Land would avert a total of $800,000 of private expenditures, 40 % of which would be averted within the poorest income quintile.

### Estimating Financial Risk Protection

Once the amount of private expenditures that may be ‘crowded out’ is estimated (Sect. 2.3), ECEA attempts to ‘scale’ this amount of expenditures by disposable income at the individual level, to estimate the FRP provided by the policy. In other words, ECEA tries to account for the fact that an individual that has an annual income of $100,000 and a loss of $10 remains much less severely impacted than an individual who has an annual income of $100.

To estimate FRP, several metrics can be used including: (1) the number of cases of catastrophic health costs averted, estimating the number of individuals no longer crossing a ‘catastrophic’ threshold of income (e.g., 10, 20, 40 % of income) as a result of the costs faced [19, 26]; (2) the number of cases of poverty averted, estimating the number of individuals no longer crossing a given ‘poverty line’ (e.g., national poverty line or international poverty line of $1.90 per day as given by the World Bank [27]) as a result of the costs faced [28]; and (3) a money-metric value of insurance provided, quantifying the willingness to pay or insurance risk premiums associated with the policy [25, 29, 30].

Each metric (1-2-3) implies a mathematical formulation involving both expenditures and costs incurred by individuals and their disposable income (denoted *y* in what follows). In the estimation procedure, one should use the individual income when it is available from the data inputs; otherwise, one should construct an income distribution in the population. For example, one possibility is to use as a proxy a Gamma distribution of income in the population easily constructed from gross national income per capita and Gini coefficient [27]. Specifically, a Gamma distribution requires two parameters that can be expressed from two inputs capturing both a mean (e.g., gross national income per capita) and a dispersion (e.g., Gini). The corresponding algorithmic implementation is described in great detail elsewhere [31, 32].

The unit of analysis selected for income may be at the individual or household levels. The time frame over which the income is evaluated may be annual or another length. These choices will depend on the availability of data inputs and on the point of view of the policy examined and the policy maker.

For the estimation of FRP, the analyst should use one metric among the three distinct metrics that we now detail.

#### Cases of Catastrophic Health Costs Averted

Given a specific income threshold *Th*, a case of catastrophic health cost is counted when at the individual level we have the realization: $\left( {c_{{{\text{DM}},k}} + c_{{{\text{DNM}},k}} } \right) > y*{Th}$.

Hence, the FRP afforded by the policy will correspond to the counting of the number of cases of catastrophic health costs averted owing to the reduction in the incidence of: *i*_*k*_ * *u*_*k*_ * (*c*_DM,*k*_ + *c*_DNM,*k*_).

This corresponds to a direct comparison of the number of cases of catastrophic health costs before and after the policy, with a numerical integration along the income distribution of the population targeted.

#### Cases of Poverty Averted

Given a specific income poverty line *Pl*, a case of poverty is counted when at the individual level, we have the two realizations: (i) *y* > *Pl*, and (ii) *y* − (*c*_DM,*k*_ + *c*_DNM,*k*_) < *Pl*.

Hence, the FRP afforded by the policy will correspond to the number of cases of poverty averted owing to the reduction in the incidence of: *i*_*k*_ * *u*_*k*_ * (*c*_DM,*k*_ + *c*_DNM,*k*_).

This corresponds to a direct comparison of the number of cases of poverty incurred before and after the policy, with a numerical integration along the income distribution of the population targeted.

#### Money-Metric Value of Insurance

We can use a utility-based model where risk-averse individuals value protection from the risk of uncertain adverse events [25, 29, 30, 33–36]. We estimate the expected value of the gamble associated with the eventuality of the disease treatment with probability *i*_*k*_ * *u*_*k*_ and cost *c*_*k*_. We use a constant relative risk aversion utility function: $w\left( y \right) = \frac{{y^{1 - r} }}{1 - r}$ for *r* > 0 and *r* ≠ 1, where *r* is the Arrow–Pratt coefficient of relative risk aversion. Following a long line of literature [35–41], a coefficient of relative risk aversion *r* = 3 (high degree of risk aversion) is often used. However, opinions diverge in the literature over the value of *r* [42–47].

First, consider the scenario under uncertainty before the policy. The expected value of income to an individual who faces the gamble involving illness-related costs is expressed as:

6 $$ E\left( y \right) = i_{k} u_{k} \left( {y - c_{{{\text{DM}},k}} - c_{{{\text{DNM}},k}} } \right) + \left( {1 - i_{k} u_{k} } \right)y. $$

Second, consider the scenario under certainty, and assume the same individual’s utility can be expressed with a constant relative risk aversion utility function (see above). In this certain scenario, the ‘certainty equivalent’ for the individual, that is the income she/he is willing to have the outcome certain denoted as $y^{*}$ is given by:

7 $$ y^{*} = \left[ {i_{k} u_{k} \left( {y - c_{{{\text{DM}},k}} - c_{{{\text{DNM}},k}} } \right)^{1 - r} + \left( {1 - i_{k} u_{k} } \right)y^{1 - r} } \right]^{{\frac{1}{1 - r}}} . $$

Subsequently, the money-metric value of insurance (risk premium) at the individual level is: *E*(*y*) − y^*^ [25]. At the population level, the insurance value is obtained after including the coverage and the effectiveness of the policy and with a numerical integration along the income distribution of the population targeted.

Consider that in Land the poverty line is *Pl* = $600. Individuals in the poorest and poorer income quintiles have an income of *y* = $300 and *y* = $470, respectively; individuals in the middle quintile have an income of *y* = $640, and individuals in the richer and richest quintiles have an income of *y* = $810 and *y* = $980, respectively. In this case, only individuals in the middle quintile could fall under the poverty line as a result of TB: 3200 of them would be ‘impoverished’ by TB-related costs. Hence, with UPF for TB treatment, 3200 poverty cases (i.e., *B*_FRP_) would be averted, all being in the middle-income group.

### Quantifying the Total Costs of the Policy

ECEA calculates the total net costs owing to implementation of the policy from the perspective of the policy maker (i.e., usually the government), and these costs can notably vary by population subgroup. ECEA exactly pursues the same approach as in a traditional CEA except that it examines the net costs procured across *k* population subgroups in lieu of one overall population in the case of CEA. As in a CEA, if the intervention procured is a preventive intervention, the estimation of the net costs (costs of the intervention minus cost savings as a result of disease averted) are estimated.

In Land, UPF for TB treatment would be provided to 50 % of the TB-infected individuals at a unit cost of *c* = $100. Hence, the net costs to the government would be:

8 $$ C = 0.50*\left\{ {8000;6000;4000;2000;0} \right\}*\$ 100 = \$ \left\{ {400000;300000;200000;100000;0} \right\}. $$

The total net costs to the government would be $1 million.

## Extended Cost-Effectiveness Analysis: Findings and Interpretation

**Fig. 3:**
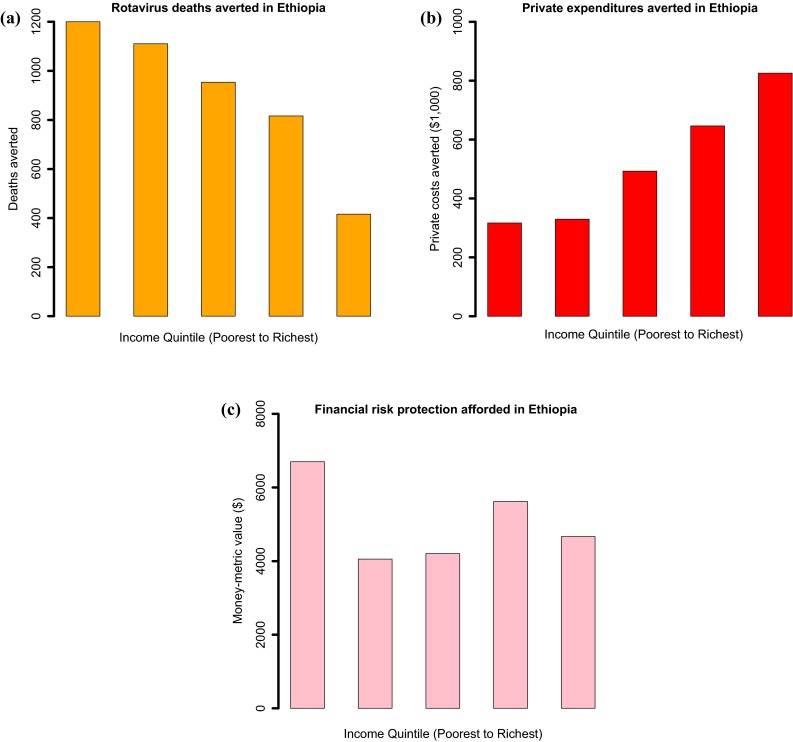
Estimated distribution across income quintiles in Ethiopia of: (**a**) rotavirus-related deaths averted; (**b**) rotavirus-related private expenditures crowded out; and (**c**) financial risk protection afforded (measured by a money-metric value of insurance), with universal public finance of rotavirus immunization. Source: based on estimates from [48]

**Fig. 4:**
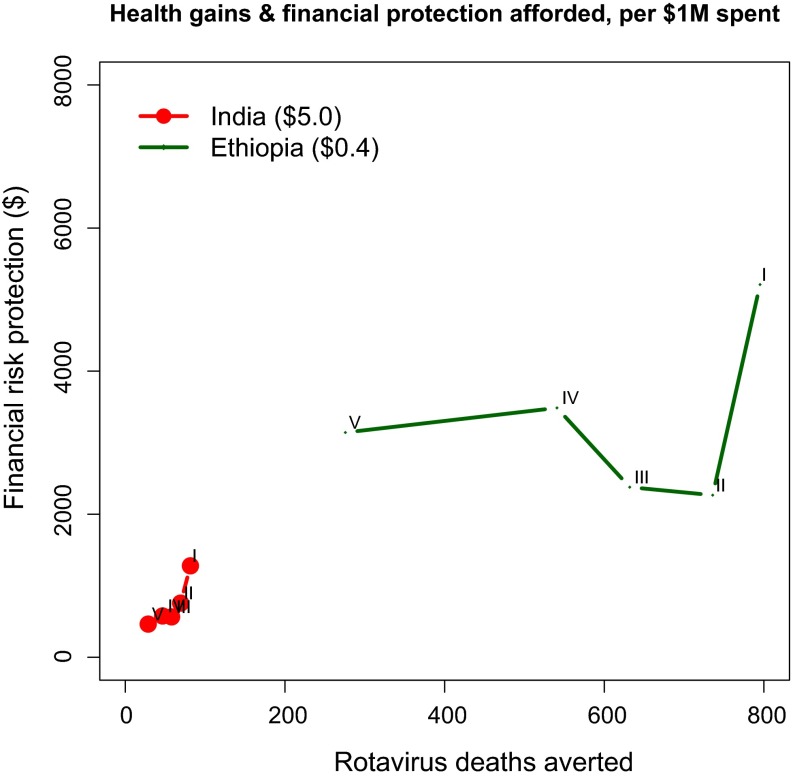
Deaths averted and financial risk protection (measured by a money-metric value of insurance) afforded with universal public finance for rotavirus immunization, per $1 million spent, India (vaccine price of $5.0) and Ethiopia (vaccine price of $0.40). Income quintiles: I = poorest, II = poorer, III = middle, IV = richer, V = richest. Source: based on estimates from [48]

ECEA examines four dimensions disaggregated per *k* population subgroups: health gains, private expenditures averted, FRP afforded, and the net costs of the policy. Usually, ECEA displays the three outcomes of health gains, private expenditures crowded out, and FRP, by population stratum (Fig. 3). Furthermore, the two major outcomes of ECEA, health gains and financial protection per population stratum, can be scaled with the net cost of the policy to a particular budget constraint or per dollar expenditure (Fig. 4). The motivation is to enable the expression of ECEA findings in terms of the ‘efficient purchase’ of financial protection and equity, in addition to the efficient purchase of health gains, as in a traditional CEA. In a practical sense, the analyst can define a financial protection incremental cost-effectiveness ratio (i.e., ICER_FRP_):

9 $$ {\text{ICER}}_{\text{FRP}} = \frac{C}{{B_{\text{FRP}} }} . $$

For instance, in the case of UPF for TB treatment, this would yield an FRP ICER of $313 per poverty case averted (from a total cost of UPF for TB of $1 million and 3200 poverty cases averted).

**Fig. 5:**
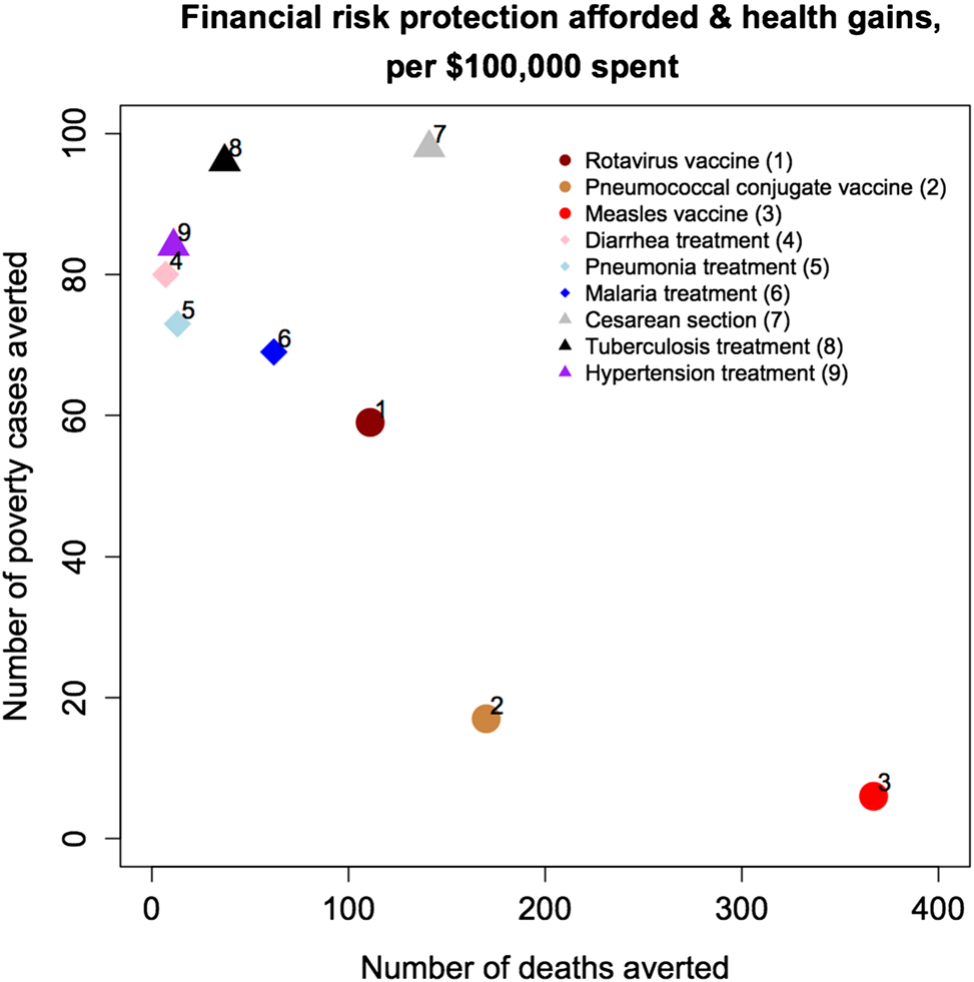
Financial risk protection afforded (poverty cases averted) vs. health gains (deaths averted), per $100,000 spent (in 2011 US$), for each of nine interventions provided through universal public finance in Ethiopia. Source: adapted from [28]

In this respect, ECEA can compare a range of policies and interventions along the two following efficiency criteria: (1) health benefits and (2) FRP (Fig. 5). In doing so, ECEA enables the inclusion of multiple criteria into the decision-making process. Importantly, it enables the design of health insurance benefits packages, based on the quantitative inclusion of information on how much FRP can be bought, in addition to how much health can be bought, per dollar expenditure on healthcare. Depending on policy makers’ and users’ preferences, one could directly select and optimize the choice of the benefits packages.

Some health policies will rank higher on health gains or financial protection relative to the other. ECEA allows policy makers to take both health and non-health outcomes into account when making decisions and thus to more effectively target scarce healthcare resources towards specific policy objectives. For example, financial protection provided through risk pooling may be the rationale to include an intervention, while a desire to increase coverage and decrease mortality may be the rationale for another. Understanding this distinction can be critical to achieve either goal. The ECEA approach also provides policy makers information on how they might sequence the development of healthcare packages as the health and financial needs of populations evolve and resource envelopes change, which is especially relevant in the context of addressing the epidemiological transition and moving toward universal health coverage [49].

Last, ECEA stresses the poverty reduction benefits of health policies. In doing so, it provides quantitative information that enables the comparison of health policy impact with other sectors outside of health (e.g., education, agriculture, transport), of particular relevance for economic development and ministries of finance in low- and middle-income countries. In this respect, ECEA can provide critical insight into how to select and sequence the health services to be provided on the path towards achievement of the sustainable development goals.

## Discussion

We presented a tutorial for conducting ECEA using a simplified example. ECEA provides quantitative methods for HPA, and further assesses the health and financial consequences of policies, including FRP and disaggregated outcomes per population stratum of interest.

The ECEA approach permits the inclusion of non-health benefits (financial protection) and distributional consequences and equity in the economic evaluation of health policies. It enables the consideration of key criteria into the resource allocation problem and into the design of the health benefits package. Focusing on three distinct outcomes and their distributions, ECEA can quantify the returns on investment (per $ spent) along the dimensions of poverty reduction, health benefits, and gains among the bottom 40 % of populations. When two policies exhibit similar returns on health benefits, ECEA can point to the policy that provides greater poverty reduction or larger improvements for the bottom 40 %. Likewise, if poverty reduction or improving outcomes for the bottom 40 % is a major policy objective, ECEA can identify the policy investments that bring the greatest impact along these two dimensions. Similarly, ECEA can explicitly point to the tradeoffs that may arise between increased health benefits and promoting FRP and equity.

ECEA studies are highly context specific and depend substantially on the local epidemiology of the setting (e.g., the endemicity and the distribution of diseases), the health system infrastructure and constraints (e.g., the presence and the distribution of health facilities), the wealth of the population (e.g., a low- vs. middle- vs. high-income country) and the underlying financial arrangements (e.g., the existence of social insurance or community-based insurance programs). Therefore, ECEA studies can be data intensive, requiring most importantly disaggregated inputs per specific population subgroups and out-of-pockets costs borne by individuals and their families. In particular, the inclusion of FRP into economic evaluations may not be so relevant in countries that have universal health insurance and where individuals are protected from medical impoverishment. Yet, in such countries, ECEA could still point to the financial protection benefits of specific interventions once being included into insurance schemes.

As mentioned previously, ECEA fundamentally builds on CEA. Therefore, the general approach to CEA including the adherence to standard health economic methods and guidelines [2, 50] remains identical. For instance, the same uncertainty analyses, such as probabilistic sensitivity analysis, can be used for ECEA as for CEA. Uncertainty in ECEA could be well characterized (e.g., with the use of 95 % uncertainty ranges) around both outcomes and distributions, aiming for example to compare uncertain policies with likely large benefits against more certain policies with likely small benefits. Yet, new issues arise with ECEA, essentially related to the illness-related costs faced by individuals and income. Most ECEAs conducted so far have restricted such private illness-related costs to out-of-pocket costs including direct medical costs and direct non-medical costs (e.g., transportation costs), owing to data availability. Yet, when data become available, indirect costs including productivity losses and wages foregone owing to illness, as well as borrowing and consumption smoothing over time, should be included. Furthermore, we did not address here the issue of the opportunity costs and assumed incremental budget money to be available, as in growing economies. Nonetheless, ECEA could well include opportunity costs (displacement of funds from existing programs), as a result of fixed budgets within the health sector or the public sector as a whole, for financial protection for example. As an illustration, one could estimate the increased taxes required for individuals to fund new interventions through public finance.

ECEA was initially developed with the case study of universal public finance for TB treatment in India [25], examining health and financial outcomes per socioeconomic group. However, ECEA is not solely concerned with socioeconomic distributions and income quintiles. Importantly, ECEA was conceived to examine any type of relevant disaggregation in a population. Regional and geographical distributions, rural and urban settings, as well as ethnic groups, sex, and marginalized populations where health and financial outcomes may vary substantially can be of critical interest and be the foci of ECEA studies. Finally, the intent of ECEA is to incorporate the quantification of non-health benefits into economic evaluations for health, and its primary non-health benefit of interest has been FRP. That being said, ECEA could well include supplemental non-health benefits such as educational benefits (e.g., school days gained through deworming policies), environmental impact, or indirect effects to relatives. ECEA initially focused on the two additional (to health benefits) dimensions of financial protection and distributional consequences, as they are two important objectives of health systems according to the World Health Organization [17].

## Conclusions

ECEA is meant for HPA, specifically to evaluate the health and financial consequences of public policies in four domains: (1) the health gains; (2) the FRP benefits; (3) the total costs to the policy makers; and (4) the distributional (e.g., across socioeconomic groups) benefits. ECEA can assess the policy impact on the prevention of medical impoverishment and the promotion of equalization of health among individuals. In this sense, ECEA focuses on the higher level of health policies (e.g., public finance, taxation), and quantitatively assesses the health and financial consequences of policies, including financial protection and disaggregated outcomes per population stratum of interest. The ECEA approach permits the inclusion of non-health benefits and distributional consequences and equity in the economic evaluation of health policies. It enables the consideration of key criteria into the resource allocation problem and into the design of health benefits packages.

## Box: Symbols used in the extended cost-effectiveness analysis tutorial

**Table:**

Symbol	Definition
HP	Health policy
*P*	Population
*P*_*k*_	Population subgroup *k*
Cov	Intervention coverage
Eff	Intervention effectiveness
*D*_*k*_	Disease burden in population subgroup *k* before policy
*i*_*k*_	Incidence of illness in population subgroup *k* before policy
*B*_*H*,*k*_	Health gains in population subgroup *k* owing to policy
*PE*_*k*_	Private expenditures incurred in population subgroup *k* before policy
*PE*_av,*k*_	Private expenditures averted in population subgroup *k* owing to policy
*B*_FRP,*k*_	Financial risk protection benefits in population subgroup *k* owing to policy
*u*_*k*_	Healthcare use in population subgroup *k* before policy
*c*_DM_	Out-of-pocket direct medical costs
*c*_DNM_	Out-of-pocket direct non-medical costs
*c*_*I*_	Indirect costs
*y*	Income
*Th*	Income threshold
*Pl*	Poverty line
*r*	Coefficient of relative risk aversion

## Footnotes

1 Note that formula (3) may be generalized to include prevention.
